# Genotoxicity assessment of a pharmaceutical effluent using four bioassays

**DOI:** 10.1590/S1415-47572009000200026

**Published:** 2009-06-01

**Authors:** Adekunle A. Bakare, Alabi A. Okunola, Olusanmi A. Adetunji, Hafeez B. Jenmi

**Affiliations:** 1 Cell Biology and Genetics Unit, Department of Zoology, University of Ibadan, Oyo StateNigeria; 2 Department of Basic Applied Science, Babcock University, Ogun StateNigeria

**Keywords:** genotoxicity, pharmaceutical effluent, mouse, *Allium cepa*, chromosome, spermatozoa, micronucleus

## Abstract

Pharmaceutical industries are among the major contributors to industrial waste. Their effluents when wrongly handled and disposed of endanger both human and environmental health. In this study, we investigated the potential genotoxicity of a pharmaceutical effluent, by using the *Allium cepa*, mouse- sperm morphology, bone marrow chromosome aberration (CA) and micronucleus (MN) assays. Some of the physico-chemical properties of the effluent were also determined. The *A. cepa* and the animal assays were respectively carried out at concentrations of 0.5, 1, 2.5, 5 and 10%; and 1, 5, 10, 25 and 50% of the effluent. There was a statistically different (p < 0.05), concentration-dependent inhibition of onion root growth and mitotic index, and induction of chromosomal aberrations in the onion and mouse CA test. Assessment of sperm shape showed that the fraction of the sperm that was abnormal in shape was significantly (p < 0.05) greater than the negative control value. MN analysis showed a dose-dependent induction of micronucleated polychromatic erythrocytes across the treatment groups. These observations were provoked by the toxic and genotoxic constituents present in test samples. The tested pharmaceutical effluent is a potentially genotoxic agent and germ cell mutagen, and may induce adverse health effects in exposed individuals.

## Introduction

Pharmaceuticals are produced and used in increasingly larger amounts every year, this having caused the industry to become one of the major contributors to industrial waste. Generally, pharmaceutical industries do not generate uniform waste streams, due to the variety of medicines produced during any given processing period (Houk, 1992). In recent times, a wide range of pharmaceuticals have been found in fresh and marine waters, and it has been shown that even in reduced quantities, some of these compounds are potentially capable of causing harm to both aquatic and terrestrial life forms (Jonathan and Nicolaos, 2005). The presence of pharmaceutical chemicals in the environment is a matter of concern due to their lipophilic and non-biodegradability nature, as well as their biological activities (Velagaleti and Burns, 2006). Currently, there is scarce of measurable evidence of the environmental impact of pharmaceutical chemicals on human health (Christensen, 1998).

A review of studies using microbial assays has shown that pharmaceutical waste does not appear to be particularly mutagenic, although it may be genotoxic by other mechanisms (McGeorge *et al.*, 1985; Moller *et al.*, 1985; Houk and DeMarini, 1988; Sanchez *et al.*, 1988). Houk (1992) suggested that, even though there is no specific evidence to suggest this, the chemical composition of pharmaceutical waste may make it unsuitable for microbial assays, since it could contain antibiotics or bacterial growth inhibitors. Thus, to be able to arrive at sufficient conclusions on the potentially genotoxic, mutagenic and masking effects of pharmaceutical waste constituents, it is important that other test systems be utilized, and also chemical characterization carried out on this group of wastes. In the present study, four eukaryotic mutagenicity assays, namely the *Allium cepa* assay, the mouse sperm morphology assay, the micronucleus (MN) test and the chromosome aberration (CA) assay in mouse bone marrow cells, were used to evaluate the genotoxic and mutagenic potential of effluents from a pharmaceutical company. These are the standard bioassays that best reflect the delicate balance between pathways for activation and inactivation of chemicals in human beings.

**Figure 1:**
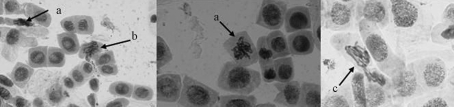
Chromosomal aberrations (arrowed) induced in *Allium cepa* root tips by the pharmaceutical effluent. (a) sticky chromosomes, (b) chromosomes with spindle disturbance, (c) disoriented chromosomes. Magnification 1000x.

**Figure 2:**
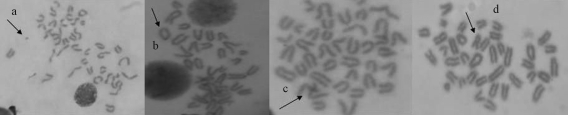
Chromosomal aberrations (arrowed) induced in bone marrow cells of mice exposed to the pharmaceutical effluent. (a) chromatid break, (b) ring chromosome, (c) chromatid exchange, (d) dicentric chromosome. Magnification 1000x.

**Figure 3:**
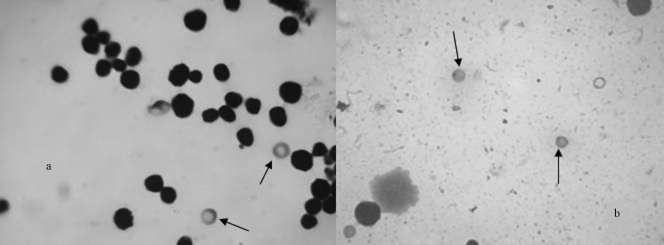
Micronuclei (MN) induced in mice exposed to the pharmaceutical effluent (a) Normal Polychromatic erythrocyte (b) Micronucleated Polychromatic Erythocyte (MNPCE). Magnification 1000x.

**Figure 4:**
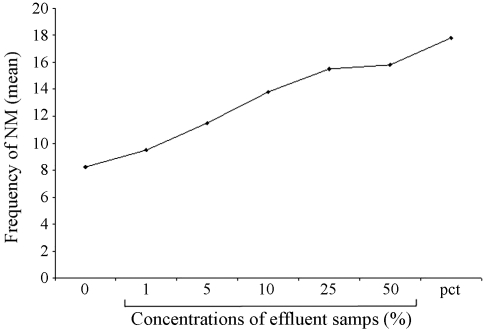
Frequency of MN induced in bone-marrow cells of mice exposed to the pharmaceutical effluent Zero: negative control (distilled water) Pct. Positive control (cyclophosphamide 20 mg/kg body weight).

**Figure 5:**
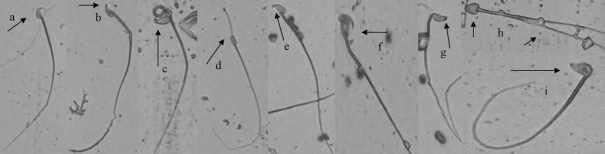
Abnormal sperm cells induced in mice exposed to different concentrations of the pharmaceutical effluent (a) normal sperm cell, (b) wrong-angled hook, (c) folded sperm, (d) pin head, (e) very short hook, (f) wrong tail attachment, (g) No hook, (h) double-tailed sperm with amorphous head, (i) amorphous head. Magnification 800x.

## Material and Methods

### Effluent collection

The raw effluent from a pharmaceutical plant in Lagos State, Nigeria was collected in two 10 L plastic containers, from the point of discharge into the environment. The company produces analgesics, anti-malarias, anesthetics, multivitamins, antibiotics, antihistamines, human vaccines, sulphonamides and antiemetics. The collected material was filtered and the pH taken, to be then kept at 4 °C until use.

### Biological materials

Onions (*Allium cepa*, L., 2n = 16, Family *Amaryllidaceae*), obtained commercially at the Bodija market, Ibadan, Nigeria, were sun-dried for 2 weeks. The dry bulbs, rotten ones excluded, were later used for the test. Young male Swiss albino mice (*Mus musculus*) of 6-10 weeks old, which had been inbred for several generations, were obtained from the animal breeding unit of the Department of Physiology, University of Ibadan, Nigeria. They were kept in a pathogen free, well ventilated animal house at the Department of Zoology, University of Ibadan, for 2 weeks in order to acclimatize. They were maintained in the same room throughout the period of this study. Food (Ladokun pelleted feed®) and drinking water were supplied *ad libitum*. The mice were divided into 3 categories for animal assays: the CA assay and MN test for mice 8 weeks old, and the sperm morphology assay for mice 12-14 weeks old.

### Physico-chemical properties and heavy metal analysis

The effluent was analyzed for a number of standard physico-chemical properties, including chemical oxygen demand (COD), total dissolved solids (TDS), alkalinity, biochemical oxygen demand (BOD), chlorides, nitrates, ammonia and phosphates, according to methods described by APHA (1998). Nine metals (including eight heavy metals) namely aluminum (Al), cadmium (Cd), copper (Cu), chromium (Cr), iron (Fe), mercury (Hg), zinc (Zn), nickel (Ni) and manganese (Mn) were analyzed in the effluent sample according to standard analytical methods (USEPA, 1996; APHA, 1998). Briefly, 100 mL of the effluent was digested by heating with concentrated HNO_3,_ and the volume reduced to 3-5 mL. This volume was made up to 10 mL with 0.1 N HNO_3_. Concentrations of the metals were estimated by using an Atomic Absorption Spectrophotometer (Perkin Eelmer E. Analyst, 2000, USA).

### *Allium cepa* test

The modified *A. cepa* assay (Fiskesjo, 1997; Bakare and Wale-Adeyemo, 2004; Babatunde and Bakare, 2006) was employed in this study. The outer scales of the onion bulbs and any brownish bottom plate were removed, leaving the ring of primordial root intact. The peeled bulbs were placed into fresh tap water during the cleaning procedure, so as to protect the primordial from drying. Thereafter, the bulbs were placed into 100 mL beakers containing 0.5%, 1.0%, 2.5%, 5% and 10% concentrations (v/v, effluent/distilled water) from the effluent. Twelve onion bulbs were set up in each concentration, out of which those 10 presenting the best root growth were selected for analysis of root growth inhibition. Distilled water was used as negative control. The experiment was performed in the dark at 27 ± 1 °C. Test liquids were changed daily. On the second day (48 h), root tips of two bulbs in the experimental and control groups were fixed in ethanol:glacial acetic acid (3:1, v/v), to be then squashed on slides for chromosomal analysis, as previously described (Bakare *et al.*, 2000; Bakare, 2002). On the third day (72 h), measurements were taken of the length of each root from the bulb, by test sample concentration. The percentage of reduction in root-growth in relation to the control and EC_50_ was obtained from the values thus come by. ASTM (1994) minimal statistical guidelines for conducting early seedling growth tests were used in the analysis of measured root length. The effects of the effluent on the morphology of growing roots were also examined.

### Sperm morphology assay

Induction of sperm abnormalities was studied according to Wyrobek *et al.* (1983) and Bakare *et al.*, (2005). Five concentrations of 1%, 5%, 10%, 25% and 50% (v/v, effluent/distilled water) of the effluent were considered together with the positive (cyclophosphamide 20 mg/kg body weight) and negative (distilled water) controls. A single intraperitoneal (IP) injection of 0.5 mL of the different test-sample concentrations was administered to the mice daily for 5 consecutive days. The IP route was favored since it is one of the fastest and most efficient means of delivering test-chemicals into test-animals in a short-term-assay. The exposure period was 35 days and 5 mice were treated for each effluent concentration. Sperm was sampled from the caudal epididymes at 5 weeks from the first injection, since spermatogenesis in mice takes 34.5 days until completion (Bartke *et al.*, 1974). The mice were sacrificed by cervical dislocation and their epididymes surgically removed. Two sperm suspensions were prepared from the cauda of each testis by mincing the cauda in physiological saline. Smears were prepared on grease-free slides after staining with 1% Eosin Y for 45 min. The slides were air-dried and coded for subsequent microscopic examination under oil immersion at 1000x. For each mouse, 800 sperm cells were assessed for morphological abnormalities according to the criteria of Wyrobek and Bruce (1975).

### Chromosome aberration assay

Five groups of mice (3 mice per group) were intraperitoneally exposed to 1%, 5%, 10%, 25% and 50% (v/v, effluent/distilled water) of test-sample concentrations for 48 h (1 mL of each concentration/mouse at 24 h intervals). Cyclophosphamide (20 mg/kg body weight) and distilled water were used as the positive and negative controls, respectively. Chromosome preparations were made as previously described (Preston *et al.*, 1987; Alimba *et al.*, 2006). Mice were injected with an aqueous solution of colchicine (2.5 mg/kg bw, IP), 2 h prior to scheduled killing by cervical dislocation. The bone-marrow cells were aspirated into 2.2% (w/v) sodium citrate and centrifuged at 2000 rpm. for 5 min. The pellets obtained were mixed in an aqueous solution of KCl (0.075 M) and left for 30 min at room temperature (29 °C). Cells were re-centrifuged, fixed in cold Carnoy fluid (methanol: glacial acetic acid, 3:1 v/v) and dropped onto clean chilled slides. Finally, the slides were air-dried and stained with 5% Giemsa (v/v, stock Giemsa stain/distilled water). The mitotic index was calculated by counting the metaphase cells from approximately 3000 cells/concentration and expressed in percent. CAs were scored blind to treatment and at least 50 well spread metaphase cells/mouse were analyzed.

### Micronucleus test

Five groups of mice (4 mice per group, 22-30 g each) were utilized in this assay, considering concentrations 1%, 5%, 10%, 25% and 50% (v/v, effluent/distilled water) of the test sample as against negative (distilled water) and positive (cyclophosphamide 20 mg/kg body weight) controls. Each mouse/group was IP exposed to 0.5 mL of each concentration for 96 h. Bone marrow preparation for micronuclei assessment was according to the procedure of Schmid (1975, 1976). Briefly, the animals were sacrificed by cervical dislocation. The femurs were removed from each and bone marrow flushed from the bones with Foetal Bovine Serum (Sigma Aldrich Cheme GmbH, Germany). Cells were centrifuged at 2000 rpm for 5 min and slides stained with May-Grunwald and Giemsa stains. At least 1000 cells/animal were scored for micronuclei in polychromatic erythrocytes (MNPCE). The differential staining of PCEs (bluish-purple) and normochromatic erythrocytes (NCEs, pinkish-orange), and the relative size of the erythrocytes, are indices for differentiating them.

### Statistical analysis

The SPSS® 14.0 statistical package was used for data analysis. Data obtained were expressed as percentage frequency and mean ± standard error (mean ± standard deviation in the CA test). Significance at the different dose-level of each assay was tested by using the Dunett t- test. Data on root-growth inhibition were expressed with 95% confidence limits and ANOVA was used for testing significance. Differences between the negative control-group and individual dose-groups were analyzed at the 0.05 and 0.001 probability levels.

## Results

Physical and chemical characteristics of the pharmaceutical effluent are shown in Table 1. The pH was 5.70, and there was an offensive odour. Chloride and nitrate levels were very high, likewise with Zn, Fe and Cu.

### *Allium cepa* assay

Table 2 shows the results from macroscopic and microscopic analysis of treated *Allium cepa* roots. Root growth attained a maximum in the control (distilled water). Here, the roots were whitish in color, elongated and straight, with no morphological deformities. At the various concentrations of the test sample, there was a gradual statistically significant (p < 0.05) concentration-dependent inhibition of root growth. The least mean root growth and the highest mean root growth were obtained at the 5% and 0.5% concentrations, respectively. There was no root growth at a concentration of 10%. Morphological deformities such as very short, bent, spiral and crochet-like roots were also observed at tested concentrations, especially at a concentration of 5%. The EC_50_ value obtained from the % inhibition value was 1.82%. Under microscopic analysis, there was a concentration-dependent reduction in mitotic index, compared to the negative control value of 33.5%, in all concentrations. Chromosomal aberrations (Figure 1a-c) were induced in all the different concentrations, all (except at the 0.5%) being statistically significant (p < 0.05).

### Chromosome aberration assay

Exposure of mice to the effluent sample for 48 h inhibited MI in bone-marrow cells in a dose-dependent manner, but this was only statistically significant (p < 0.05) at the 10, 25 and 50% concentrations of the test sample (Table 3). Different types of dose-dependent and statistically significant (p < 0.05) CAs were observed (Figure 2a-d).

### Micronucleus test

Figure 3 shows the micronuclei induced in the bone marrow cells after exposure of mice to the test sample. Compared with the negative control, there was a statistically significant (p < 0.001), dose- dependent increase in MN at all concentrations, except 1% and 5% (Figure 4). The dose-response equation for this effect is y = 7.45 + 1.65x (y = 0.99).

The positive control induced a significant induction of CA and MN in positive control groups.

### Sperm morphology assay

Figure 5 (a-g) shows the different types of abnormal sperm cells observed, 5 weeks from the 1^st^ day of exposure of male mice to the test pharmaceutical-effluent. The frequency of abnormal sperm cells in the negative control was 9.85%. There were 27.8%, 31.0%, 33.3%, 40.6% and 47.2% of abnormal sperm cells at the tested concentrations of 1%, 5%, 10%, 25% and 50%, respectively. This induction of abnormalities was statistically significant (p < 0.05) and concentration-dependent at all concentrations except at 1%. Generally speaking, sperm with wrong-angled hooks (Figure 5b) were the most prominent (26.1%), whereas double-tailed sperm cells (Figure 5h) were very few in number (0.6% occurrence).

## Discussion

Industrial discharge is recognized as one of the major sources of toxic chemicals in the environment. In the present study, the genotoxicity and mutagenicity of a pharmaceutical effluent was assessed by using a battery of *in vivo* assays in plant and animal systems. The results of the physico-chemical characteristics of the effluent showed the presence of certain sample-constituents at concentrations beyond the permitted limits set by international regulatory authorities (Table 1). Heavy metal analysis of effluent-samples showed the presence of Cu, Ni, Cr, Cd, Mn, Al, Fe, and Zn at various concentrations. These metals have the potential to induce mutation and cancer in living cells. A mixture of these may result in synergistic chemical combinations that are more harmful than the individual constituent itself. Studies in experimental animals indicate that Ni (Haugen *et al.*, 1994) and Cd (Elinder and Jarup, 1996) are carcinogenic. Hexavalent Cr was reported to have induced chromosomal aberrations, and micronuclei and single-strand breaks in mammalian cells (Wise *et al.*, 2002), besides gene mutation in bacteria (DeFlora *et al.*, 1990). Trivalent Fe was also reported as having been responsible for the high level of micronuclei in newt larvae (Godet *et al.*, 1996). The exposure of mice to Zn results in both single-strand breaks in DNA, as measured by the comet assay (Banu *et al.*, 2001), as well as chromosomal aberrations (Gupta *et al.*, 1991). Ni is known to produce highly selective damage to heterochromatin (Costa *et al.*, 1994). It has also been reported that Cd, Cu and Fe gave rise to reactive oxygen species in eukaryotic systems (Ghio *et al.*, 2002; Radetski *et al.*, 2004). The constituents analyzed herein do not represent all, or even most, of the chemicals that could have been included in the test-sample. An effluent is a complex mixture of organic and inorganic chemicals, and of many unidentified toxicants known as non-conventional pollutants (NCPs), all of which may pose risks of an unknown magnitude to humans.

From data obtained through bioassays, it is shown that the tested effluent is cytotoxic, mutagenic and genotoxic. The results of the *A. cepa* assay are indicative of a linear relationship between macroscopical (root-growth inhibition) and microscopical (genotoxicity) parameters. The macroscopic effect appears to be the most sensitive parameter, since any genotoxic effect manifest in a test sample, either directly or indirectly, is likely to result in inhibition of growth (Fiskesjo, 1997). The EC_50_ value indicated that the tested sample is highly toxic, with the highest tested concentration (10%) totally inhibiting root-growth in the treated bulbs. Microscopic examination allowed for assessing chromosome damage and cell-division disturbances, thus providing additional information regarding severity or mechanism of the toxic effect or of potential mutagenicity. In *A. cepa*, whenever chromosome aberrations occurred, there were almost always certain growth restrictions (Fiskesjo, 1997). This became evident in this study, and corroborates previous observations on this assay, when done in our laboratory (Bakare and Wale-Adeyemo, 2004; Babatunde and Bakare, 2006). Similar observations were also reported on dye wastewater (Somashekar and Gowda, 1983), sugar-cane factory wastewater (Mishra, 1993) and carbonaceous sugar-mill effluents (Kumar, 2000), when using the *A. cepa* test.

In the sperm morphology assay, the criteria for a positive response were satisfied: there was an increase in abnormal sperm morphology to at least double the negative control level (at all treatment levels). There was also evidence of a concentration-dependent increase in the number of aberrant sperm cells. Sperm morphology tests provide a direct measure of the quality of sperm production in chemically treated animals. Studies evaluating the genetic consequences of chemically induced sperm changes have mainly focused on understanding the genetic basis of chemically shaped abnormalities in mice. A number of lines of evidence suggest that an induced change in sperm morphology is reflected by genetic damage in the male germ cell (Topham, 1980). Wyrobek *et al.* (1983) also noted that when male germ cells are exposed *in vivo* to a test sample, a positive result demonstrates the sample ability to damage spermatogenesis. Our observations are in accordance with those of Muller and Kasper (2000) who showed that pharmaceuticals are not only capable of inducing abnormal sperm cells, but are also carcinogenic in mice. Similarly, Caldwell (1993) concluded that pharmaceutical compounds are potentially genotoxic to mouse sperm cells, and therefore suggested that the waste from such products may be equally dangerous. Thus, the sperm abnormalities observed herein are an indication that the effluent chemical constituents, in fact, exerted an effect on sperm from treated spermatogonial cells. This goes to show that the effluent constituents, in this case, were capable of interacting with the genetic processes involved in spermatogenesis in mice.

Results from the MN and CA assays showed that pharmaceutical effluents caused a decrease in MI, and induced high CA and MNPCE frequency in mouse bone-marrow cells. Marked inhibition of MI indicates test-effluent cytotoxic potentiality in mice. In the test-sample, the possible mitotic inhibition mechanism is the most likely action in microtubule functioning and/or formation (Stehrer-Schmid and Wolf, 2005). The induction of various types of structural CA elicits the clastogenic potential of pharmaceutical effluents, which, through long-term exposure, can cause somatic mutation. Increased frequency in the occurrence of acentric type aberrations indicates microtubule distortion, similar to that observed in the *A. cepa* assay in the form of chromosomes with disturbed spindle. A possible mechanism for the induction of anomalies in mouse bone-marrow cells is that the effluent was absorbed into the cells and caused change in pH within and outside cells, which might affect the activities of enzymes and the structure of DNA (Meng *et al.*, 2002). Other possible mechanisms might have been the formation of free radicals, either through auto-oxidation, pharmacodynamics or enzyme-catalyzed oxidation of electrophilic components of the effluent. Free radicals, endogenously generated on exposure to the effluent, could react with the lipid content of the cell membrane, thus resulting in lipid peroxidation in the tissues, thereby causing breakage of the DNA chain by oxidating the base component of the membrane. The free radicals could also have reacted with those protein-enzymes involved in the DNA repair mechanism, the alteration of repair enzyme activity resulting in increased frequency in DNA damage. It is important to note that the reactions of DNA and the free radicals, especially the reactive oxygen species, result in the production of covalently modified bases known as DNA adducts, which are mutagenically potent contributors to the aetiology of genetic diseases (Marnett and Burcham, 1993).

The CA and MN assays were primarily devised for evaluating the ability of test-chemicals to induce structural and/or numerical chromosomal damage. There is a correlation between the two assays. Generally, micronuclei are forms resulting from the aggregation of whole chromosomal or chromosome/chromatid fragments, aberrations and disturbances in the mitotic process (Grover and Kaur, 1999). Both types of damage are associated with the appearance and/or progression of tumours, and adverse reproductive and developmental outcomes (Krishna and Hayashi, 2000).

The genotoxicity of industrial effluents has been extensively assessed (Houk and DeMarini, 1988; Houk, 1992; Snyder and Green, 2001; Babatunde and Bakare; 2006; Siddique *et al.*, 2008). Our study provides additional and original *in vivo* information on existing *in vitro* studies on the genotoxicity of pharmaceutical effluents. The mechanism of induction of genetic damage in the four assays may be as previously described for similar complex mixtures (Alimba *et al.*, 2006; Li *et al.*, 2006a; 2006b; Bakare *et al.*, 2007). Our findings are of importance as chemically induced genetic damage has been implicated in the aetiology of many genetic diseases. The increased genetic damage caused by industrial effluent/waste mixtures in eukaryotic cells indicates a potential genetic hazard. This is of great importance to public health, seeing that environmental waste management in many developing nations is wholly inadequate.

## Figures and Tables

**Table 1:** Physico-chemical characteristics of the pharmaceutical effluent assessed for genotoxicity.

Parameters*	Effluent sample	FEPA^a^	USEPA^b^
Colour	Pale brown	-	-
pH	5.7	6-9	6.5-8.5
COD^c^	147. 04	50	410
BOD^d^	48.13	50	-
TDS^e^	336	2000	500
Salinity	524	-	-
Alkalinity	120	250	20
Hardness	80	-	0-75
Chloride	5240	-	250
Nitrate	61.3	20	10
Phosphate	42	5.0	-
NH_3_	23.4	0.01	0.02
Cd	0.002	0.01	0.005
Cr	0.01	0.05	0.10
Cu	1.02	0.01	1.0
Fe	1.80	0.3	0.30
Mn	0.46	0.05	0.05
Ni	0.020	0.05	-
Zn	10.20	5.0	5.0

*All values are in mg/L except pH and salinity (ppt.). ^a^FEPA: Federal Environmental Protection Agency (2001). ^b^USEPA: United States Environmental Protection Agency (1989). ^c^COD: Chemical oxygen demand. ^d^BOD: Biochemical oxygen demand. ^e^TDS: Total dissolved solid.

**Table 2:**
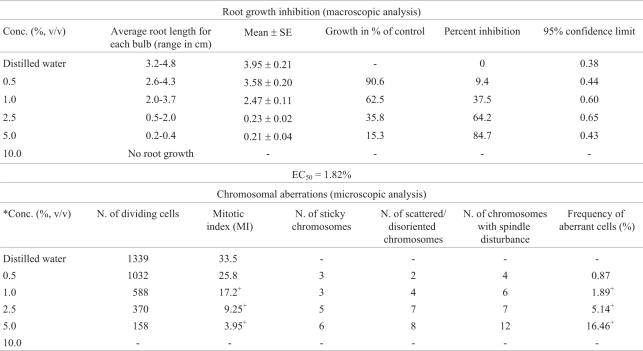
Inhibitory and cytological effects of the pharmaceutical effluent on *Allium cepa* root.

**Table 3:** Chromosome aberrations (CAs) induced in bone marrow cells of mice exposed to different concentrations of the pharmaceutical effluent.

Conc. (%, v/v)	N. of cells at metaphase	MI ± SD	Total n. of CA	Mean of CA ± SD	Frequency of aberration
Distilled water	128	4.27 ± 2.08	4	1.33 ± 0.58	2.7
1.0	123	4.1 ± 1.0	22	10.7* ± 0.58	14.7
5.0	112	3.7 ± 1.53	39	13.0* ± 2.31	26.0
10	104	3.5* ± 2.52	41	13.7* ± 3.06	27.3
25	95	3.2* ± 3.21	48	16.0* ± 4.58	32.0
50	87	2.9** ± 1.0	53	17.7* ± 5.7	35.3
Cyclophosphamide^#^	71	2.4** ± 2.65	59	19.7** ± 1.52	43.3

*p < 0.05, ** p < 0.001: levels of significance of chromosome damage in bone-marrow cells of mice *vs.* distilled water (negative control). MI: Mitotic Index (3000 cells/concentration). ^#^: 20 mg/kg body weight.
